# Supplementary material for: Improved log-Gaussian approximation for over-dispersed Poisson regression: Application to spatial analysis of COVID-19
Source: PLoS One. 2022 Jan 7;17(1):e0260836. doi: 10.1371/journal.pone.0260836 (PMC8741021; doi:10.1371/journal.pone.0260836)
Supplement: S1 File — (DOCX) [file pone.0260836.s001.docx]

**Supporting information**

**Appendix S1: Monte Carlo experiments assuming six explanatory variables**

This section compares the estimation accuracy assuming seven explanatory variables. The simulated count data $y_{i}$ is generated from the following regression:

| $y_{i}\sim odPoisson\left( \lambda_{i},\sigma^{2} \right), \lambda_{i}=\exp\left( \beta_{0}+\sum_{k=1}^{6} x_{i,k}\beta_{k} \right),$ | (S1) |
| --- | --- |

where $x_{i,p}\sim N(0,1)$. The coefficients $\beta_{1}=\beta_{2}= \beta_{3}=2.0$ and $\beta_{1}=\beta_{2}= \beta_{3}=0.5$. The RMSE values are compared across models while varying $\beta_{0}\in\{-2, -1, 0, 1, 1\}$, $\sigma^{2}\in\{1, 5\}$, and $N\in\{50, 200\}$. In each case, the simulations were iterated 500 times.

Figures S1 and S2 compare the RMSE values in cases assuming $\sigma^{2}=1$ (equi-dispersion) and $\sigma^{2}= 5$ (over-dispersion) respectively. Proposed outperform LogLinear and Taylor approximations across cases and compatible Poisson and odPoisson models. These results are consistent with the results assuming three explanatory variables (see “Case 1: Basic over-dispersed Poisson regression model”).

**Figure S1: RMSE of the coefficients in cases with seven explanatory variables (**$\boldsymbol{\sigma}^{\boldsymbol{2}}\boldsymbol{=1.0}$**)**

**Figure S2: RMSE of the coefficients in cases with seven explanatory variables (**$\boldsymbol{\sigma}^{\boldsymbol{2}}\boldsymbol{=5.0}$**)**

**Appendix S2: Monte Carlo experiments assuming group-wise random effects**

This section examines the estimation accuracy of the proposed model, assuming a Poisson MEM with group-wise random effects. The synthetic data is generated from an over-dispersed Poisson MEM defined as

| $y_{i}\sim oPoisson\left( \lambda_{i},\sigma^{2} \right), \lambda_{i}=\exp\left( \beta_{0}+x_{i,1}\beta_{1}+x_{i,2}\beta_{2}+g_{i} \right),$ | (S2) |
| --- | --- |

where $\left\{ \beta_{1},\beta_{2} \right\}=\{2, 0.5\}$ and $\sigma^{2}=5$. $g_{i}$ represents the group-wise random intercept. Samples were randomly assigned to three groups for *N* = 50 (16.7 samples per group) and 10 groups for *N* = 200 (20 samples per group). The value of $g_{i}$ was sampled from a standard normal distribution.

Following the “Monte Carlo experiments” section, the Poisson MEM (Poisson), an over-dispersed Poisson MEM (odPoisson), the Taylor approximate Poisson MEM (Tayor), and our approximation (Proposed) are estimated, and their estimation accuracies are compared while varying $\beta_{0}\in\{-2, -1, 0, 1, 1\}$ and $N\in\{50, 200\}$. In each case, the models were estimated 500 times.

Figs S3 and S4 summarize the estimated RMSEs and biases. Proposed tends to have smaller RMSEs than Poisson, odPoisson, and Taylor in cases with small samples (*N* = 50) while as small as Poisson and odPoisson in cases with *N* = 200. The estimation bias of Proposed is also as small as Poisson and odPoisson. Fig S5 compares SEs. The result is consistent with another simulation assuming spatial dependence (see the Monte Carlo experiment section); the SEs estimated from Proposed tends to be similar to odPoisson, especially when $\beta_{0}$ is large. Regarding the group effects estimates (see Fig S6), the accuracy of Proposed is slightly better than odPoisson.

Overall, the results suggest that the proposed method accurately approximates the over-dispersed Poisson MEM with group effects.

**Figure S3: RMSE of the regression coefficients (model with group effects)**

**Figure S4: Bias of the regression coefficients (model with group effects)**

**Figure S5: Means of the coefficient standard errors (**$\boldsymbol{N=200}$**)**

**Figure S6: RMSE of the estimated group effects**
