# Supplementary material for: Improved log-Gaussian approximation for over-dispersed Poisson regression: Application to spatial analysis of COVID-19
Source: PLoS One. 2022 Jan 7;17(1):e0260836. doi: 10.1371/journal.pone.0260836 (PMC8741021; doi:10.1371/journal.pone.0260836)
Supplement: Attachment — Submitted filename: response_v3.docx [file pone.0260836.s002.docx]

| **Comments from the editor** | |
| --- | --- |
| Comment | Response |
| **[E-1]** Improve clarity of the manuscript | We entirely checked the draft and revised to improve the clarity of the manuscript partly based on reviewer comments. |
| **[E-2]** Clarify why the reported main motivation is improved efficiency and accuracy for Bayesian Poisson regression but Bayesian modelling does not appear anywhere in the paper | We agree with this comment. Our approximation is not directly related to Bayesian modeling. The introduction section (lines 32-71) is revised to not to emphasize Bayesian modeling. Besides, we revised our motivation as follows (line 70-71):  We develop a closed-form approximation for the over-dispersed Poisson regression, that has (i)–(iii) and overcomes (iv)–(v). |
| **[E-3]** Clarify the meaning of "posterior variance" etc. | The term “posterior variance” was a misuse. Based on a comment from reviewer 2 (**[2-3]**), “posterior variance” is replaced with “an approximate variance for a log-transformed Poisson random deviate” (see line 93) |
| **[E-4]** Explain and motivate mismatch between the model used to simulate data and the model used to analyse it | Based on this comment and comment **[2-22]**, we now use the same model (the generalized additive mixed model implemented in the mgcv package) in the simulation and data analysis sections (see lines 238 and 319). |
| **[E-5]** Clarify the reason for the “extra” 0.5/(Y+0.5) in (6) | Thank you for the very important point.  In the revised draft, we modify our approximation by combining two approximations for non-zero counts and zero counts (see lines 74-130). As a result, we have an additional term 0.5r/(Y+0.5), which is in $y_{i}^{+}=\frac{y_{i}+0.5}{z_{i}}\exp\left( -\frac{1+0.5r}{y_{i}+0.5} \right)$ in line 128, where r is the ratio of zero counts in the explained variables. See lines 74-130 for further details. |
| **[E-6]** Revise terminology (e.g. zero-inflated Poisson, posterior variance, etc) | Terminology is entirely checked and revised mainly following comments from the reviewers. |
| **[E-7]** Poisson regression with random effects/spatial effects is already very well developed, so approximations such as this one are not strictly necessary. Please demonstrate that the proposed solution is much more efficient. | We newly explain that our approximation is advantageous in terms of (i) stability/identifiability and (ii) computational efficiency.  Regarding (i) the stability, the following sentence is added (line 56-60):  The maximum likelihood estimates of the conventional Poisson regression are unidentifiable or identifiable only weakly for certain data configuration (Silva and Tenreyro, 2010; Correia et al., 2019). As we will illustrate later, this property considerably worsens the accuracy of Poisson regression estimates especially for small samples with many zeros. Their linear approximations are free from such difficulty and can be more stable.  After that, we showed in Figures 3-4, 6-7 that our approximation tends to have smaller RMSE and bias values for small samples (N=50) with many zeros (small β_0_).  Regarding (ii) computational efficiency, we newly compare computation time in the “Computation time comparison” section (line 269-279), and show that our approximation is considerably faster than the conventional Poisson regression. |
| **[E-8]** Please clarify the meaning of, e.g., "posterior variance". In a Bayesian setting "prior" and "posterior" usually refer to distributions of the parameters. So when talking about a Poisson distribution, the "posterior" would be the a distribution of lambda (given one or more observations y), not of the Poisson RV "y" as apparently used in the paper. (See for example https://stats.stackexchange.com/a/26225). | The term “posterior variance” was a misuse. Based on a comment from reviewer 2 (**[2-3]**), “posterior variance” is replaced with “an approximate variance for a log-transformed Poisson random deviate” |
| **[E-9]** We note that Figure(s) 10 and 13 in your submission contain map images which may be copyrighted. | The figures are new and not copyrighted. |
| **[E-10]** Please include the data sources used in the Data availability statement and Methods section. We note that the source of the COVID-19 data appears to be unclear in the Methods section. Please also indicate in the Data availability statement whether you are able to openly share the code used, and if so, where others can access this. | We added the following Data availability statement (line 378-380).  Data availability statement  The code for the proposed method is available from the corresponding author on request. The COVID-19 data can be purchased from JX Press Corporation (https://jxpress.net/). |

| **Comment from reviewer 1** | |
| --- | --- |
| Comment | Response |
| **[1]** The authors need to used more than 2 explanatory variables. For example, p=4 and 8. | Thank you very much for the comment. We newly performed simulation experiments assuming 6 explanatory variables (p=6) in Appendix. S1 (see the supplement file), and verified that the result is consistent with the result when assuming 2 explanatory variables. |

| **Comments from reviewer 2** | |
| --- | --- |
| Comment | Response |
| **[2-1]** Modern computing has made it possible to model generalized linear mixed models, including models with spatial and temporal correlation. So approximations such as yours are not strictly necessary. You suggest that your approximation is a more computationally efficient. I agree this is likely, but you never demonstrate how much more efficient. | Thank you very much for the suggestive comment. We newly explain that our approximation is advantageous in terms of (i) identifiability/stability and (ii) computational efficiency.  Regarding (i) the identifiability, the following sentence is added (line 56-60):  The maximum likelihood estimates of the conventional Poisson regression are unidentifiable or identifiable only weakly for certain data configuration (Silva and Tenreyro, 2010; Correia et al., 2019). As we will illustrate later, this property considerably worsens the accuracy of Poisson regression estimates especially for small samples with many zeros. Their linear approximations are free from such difficulty and can be more stable.  After that, we showed in Figures 3-4, 6-7 that our approximation tends to have smaller RMSE and bias values for small samples (N=50) with many zeros (small β_0_).  Regarding (ii) computational efficiency, we newly compare computation time in the “Computation time comparison” section (line 269-279) and show that our approximation is considerably faster than the conventional Poisson regression. |
| **[2-2]** I don’t follow where the “extra” 0.5/(Y+0.5) in (6) comes from (lines 92-93). If Z ~ logN(m, v), by definition log Z ~ N(m,v). L 92 looks like you are computing E Z = exp(m + v/2) so the “extra” 0.5/(y+0.5) is v/2. But, the expectation in L 92 is not the equivalent of E Z, it is the equivalent of E log(Z), which = m. Said another way, It looks like you are computing log E Z, which does not equal E log Z. You want E log Z to get to equation (6). The choice of constant in log(Y+c) does matter. One example, where X is transformed, is Ekwaru and Veugelers, 2018, Stat BioPharm Res, 10:26-29. | Thank you for the very important point.  In the revised draft, we modify our approximation by combining two approximations for non-zero counts and zero counts (see lines 74-130). As a result, we have an additional term 0.5r/(Y+0.5), which is in $y_{i}^{+}=\frac{y_{i}+0.5}{z_{i}}\exp\left( -\frac{1+0.5r}{y_{i}+0.5} \right)$ in line 128. where r is the ratio of zero counts in the explained variables. See lines 74-130 for further details. |
| **[2-3]** I also could not follow or do not agree with your variance manipulations. This has two aspects. 1) Line 79 gives, according to your text, an approximation of the “posterior variance of a Poisson distribution”, citing El-Sayyad 1973. I did not read El-Sayyad and do not understand what you mean by “posterior”. Is 1/(y+c) is an approximate variance for a Poisson random deviate or an approximate variance for a log transformed Poisson random deviate? | We agree. Following this comment, we made the following revision (line 93):  Previous version: 1/(y+c) is the posterior variance of a Poisson distribution  Revised version: 1/(y+c) is an approximate variance for a log-transformed Poisson random deviate. |
| **[2-4]** 2) There appears to be a mismatch between your OD variance model and the mechanism used to simulate OD Poisson data. It was not clear in the main text how simulated from an OD Poisson distribution. I finally found the answer in Appendix S1, where you simulate Y ~ Pois(lambda), log lambda = mu + Z, Z ~ N(0, sigma^2). That generates observations from a log normal Poisson mixture for which variance Y = mu + (exp(sigma^2)-1) mu^2. You give your OD variance model as Var log Y = sigma^2/(y+c) approximately. That is not the same variance model as the variance pattern of your simulated data. Applying the Jacobian of the log Y transformation to the log normal variance, you get Var log Y approx. = 1/mu + (exp(sigma^2) -1). I presume El-Sayyad then uses a plug-in estimate of mu to estimate 1/mu as 1/(y+c). These two expressions are the same for Poisson data, i.e. when sigma^2 = 0, but not the same for OD Poisson data. | Sorry for our unclear explanation. Z ~ N(0, sigma^2) is used only for generating random intercepts in appendix, and it is not used in the other sections.  We newly added the following sentence explaining how to simulate Y (lines 185-188):  Over-dispersed Poisson distribution, which is characterized by the mean and variance, does not have probability mass function (Wedderburn, 1974). Therefore, following Gsteiger et al. (2013), $y_{i}$ is sampled from a negative binomial distribution such that the assumption of the over-dispersed Poisson distribution (mean: $\lambda_{i}$, variance: $\lambda_{i}\sigma^{2}$) is fulfilled.  The expectation and variance of the sampled data are the same as the over-dispersed Poisson distribution, which we assumed. |
| **[2-5]** You emphasize the computational efficiency. Please provide data on that. Compare the time to fit a model using a log-Gaussian approximation to the time required to fit a Poisson or OD Poisson model. The OD Poisson model could be fit by adding an observation specific random effect (to generate a log-normal Poisson distribution). | We newly compare the computation time in the “Computation time comparison” section (line 269-279). The result confirms that that our approximation is considerably faster than the conventional Poisson regression. |
| **[2-6]** Please give this a careful read for English usage and word choice. Three examples are line 33, I presume you meant “collected” not “corrected”, the usual interpretation of “zero-inflated”, and L 226, a “decade” is ten years. There are others throughout the manuscript. | Thank you very much for the comment. The three parts are revised   - collected -> corrected - zero-inflated -> removed from the draft - decades -> 10-year age groups   In addition, we entirely checked our English usage and word choice. |
| **[2-7]** L 45. I don’t understand the “lack of contiguity”. In the usual setup for Poisson count data with latent variables, the Poisson distribution is the data model conditional on the latent observation-specific mean. That latent variable has support on the non-negative half line, so a log-normal or gamma distribution for the latent variable is completely compatible. I believe you’re thinking about approximating a discrete Poisson distribution by a continuous distribution, which has nothing to do with Bayesian Poisson regression. | We apologize for our wrong word choice. “lack of contiguity”, which was a typo, is replaced with “lack of conjugacy” which we meant.　Because the log-normal distribution is not conjugate with Poisson distribution, we believe the statement is correct. |
| **[2-8]** L 75: Do you mean “prior” here or are you really talking about random effects? | The unclear part was removed. |
| **[2-9]** L 99, 108: “sample weight” has two contrasting uses: Variance = weight * constant and weighted SS = Sum weight * (y - \hat Y)^2. The weighted SS use is more frequent, because that’s how weights are specified in most (all?) software. You’re using the first definition. I suggest you explain carefully, avoiding the term weight, if you retain 1/(y+0.5). Or, define the weight as y+0.5. | We agree with this comment. The weight is now defined as y + 0.5 (e.g., the last row of Table 1; line 169). |
| **[2-10]** L 101: If the “extra” 0.5/(y+0.5) in (6) is correct and your variance model is assumed corrected, notice that adding overdispersion will change the “extra” term to 0.5 \sigma^2/(y+0.5). The weights won’t change because they are unaffected by a constant multiplier to the variance. | We agree with this comment. The following sentence is added to explain that our model is not derived from the over-dispersed Poisson model and that Monte Carlo experiments are performed to check if our model can approximate the over-dispersed model (line 136-139):  Eq. (9) approximates the Poisson regression model and controls over/under-dispersion using the $\sigma^{2}$ parameter just like the over-dispersed Poisson model. Still, because Eq. (9) is not derived from the over-dispersed model, it is unclear if our approach accurately approximates it. We examine it through Monte Carlo experiments later. |
| **[2-11]** Table 1. It would be useful to add your overdispersed approximation to this table. | Thank you for the comment. Because all the linear approximation listed in Table 1 implicitly approximate an over-dispersed/under-dispersed Poisson model, we just add the following sentence in the caption (line 167-169).  As with our model, the other models estimate the $\sigma^{2}$ parameter, which controls over-/under-dispersion, when fitting them on real data. |
| **[2-12]** L 142. 500 simulated data sets seems rather small to provide a reasonably precise estimate of the RMSE, especially when it seems some estimates are “wild”. Please either provide an estimate of the Monte-Carlo variance to demonstrate that your results from 500 simulations are sufficiently precise or increase the number of simulations (1000, or substantially more if the distribution of estimates is very skewed). | Thank you for the comment. The number of simulations is now increased to 1000. We think the results are now smooth enough. |
| **[2-13]** L 148: Please be careful about your terminology. Equation (9) is not generating zero-inflated Poisson data. It is generating data from a Poisson distribution with mean close to 0 so there is a high probability of observing 0. A zero-inflated Poisson model is a mixture model. One mixture component is a point mass at 0, with a mixture probability of p0. The second mixture component is a draw from a Poisson distribution, with mixture probability of 1-p0. Similar issue on l 227. The counts may be zero inflated, but reporting the %0’s doesn’t tell you that. | Thank you very much for the suggestive comment. We agree. The term “zero-inflated” is removed throughout this draft. |
| **[2-14]** L 181. Please cite one of the papers describing mgcv instead of linking to the CRAN page. See the information provided by citation(‘mgcv’). | The link is now replaced with Wood (2011), which is provided by citation(‘mgcv’) (line 238). |
| **[2-15]** L 192. Small se’s are good only when they still correctly describe the uncertainty in the estimate. One way to check this is to compare (i) the average estimated variance of a coefficient = average squared standard error to (ii) the observed variance of an estimate over simulations. | Thank you for the suggestive comment. Based on the comment that “small se’s are good only when they still correctly describe the uncertainty in the estimate,” the following part, which was overstated, was revised as follows:  Previous version: The proposed method stabilizes it and make the SEs small.  Revised version: The proposed method has smaller SE estimates (line 249-250).  In addition, we tried to compare (i) and (ii). Because the conventional linear regression model estimates coefficient standard errors under the assumption that the covariates are given, we attempted to evaluate (ii) the variance of an estimate over simulations while assuming the covariates as given. However, the simulation result changes depending on the covariate values, and stable results were not obtained. Because of the reason, we did not compare (i) and (ii). |
| **[2-16]** L 221/2, Figures 10 and 11. The figure numbering seems reversed from what is described in the text. | We confirmed that the numbering is correct. |
| **[2-17]** L 225: “generation” – please be consistent in your choice of names. I believe this is what was called wave in lines 216-217. Ahh (based on text later on): generation is age group. | Yes. This “generation” means age group. For clarity, “by generation” is now replaced with “by 10-year age groups” (line 303) |
| **[2-18]** L 225: Please emphasize that you are doing completely separate analyses for each wave of data. One could look at “determinants within each wave” by fitting a single model with a wave*determinant interaction. | The following sentence is added to emphasize that (line 309):  The model is fitted for each of the three datasets completely separately. |
| **[2-19]** L 225: determinants (plural) seem an overstatement. Your model only looked at pedestrian density. | We agree. The term “determinants” is replaced with “influencing factor” (line 289), which seems more appropriate. |
| **[2-20]** L 226: This is spatio-temporal data. Please explain carefully what data were used in the analysis. The text says “every decade”. Was this one day’s data, the sum over a series of days within “the decade”, all the daily observations within “the decade”, or something else? | Something else. Our data is a spatiotemporal data (time index =date). In each day, we have data by prefecture by 10-year age groups.  The term “decade”, which was confusing, is removed. The following sentence is added to clarify the definition of the data (line 302-303):  The explained variables are the number of daily cases by prefecture by 10-year age groups ( -19, 20-29, …, 70-79, 80- ). |
| **[2-21]** L 237 – 239: The spatial component of the data is per prefecture. Why include both prefecture and the spatial random effect in the model? Prefecture will capture the only spatial aspect of the data, so I don’t see where there is residual spatial dependence. | The following sentence is added to explain why we include the spatial random effect (line 316-319).  In considering countermeasures, it is important to reveal not only patterns by prefectures but also patterns across prefectures. To estimate it, we include a low rank Gaussian process $s_{i}$ which smoothly varies depending on geographic coordinates; we use the geographic center of each prefecture for the modeling. |
| **[2-22]** L 240: Why use the spmoran package instead of gamm() in mgcv? GAMM is the spatial method you evaluated in the simulation. If you need to use Moran Eigenvector Maps to model the spatial dependence, please explain the reason | Thank you for the comment. We now use the mgcv package throughout the draft. |
| **[2-23]** Poisson regression with random effects/spatial effects is already very well developed, so approximations such as this one are not strictly necessary. Please demonstrate that the proposed solution is much more efficient. | Thank you very much for the suggestive comment. As commented in the response to comment **[2-1]**, our approximation is advantageous in terms of (i) identifiability/stability for small samples and (ii) computational efficiency for large samples.  Regarding (i), we newly explained in lines 56-60 that the conventional Poisson regression are unidentifiable or identifiable only weakly for certain data configuration (Silva and Tenreyro, 2010; Correia et al., 2019) while our linear approximation is free from such difficulty and more stable. Then, we showed in Figures 3-4, 6-7 that our approximation tends to have smaller RMSE and bias values for small samples (N=50) with many zeros (small β_0_).  Regarding (ii), we newly compare the computation time in the “Computation time comparison” section (line 269-279), The result confirms that our approximation is considerably faster than the conventional Poisson regression. |
| **[2-24]** Please clarify the meaning of, e.g., "posterior variance". In a Bayesian setting "prior" and "posterior" usually refer to distributions of the parameters. So when talking about a Poisson distribution, the "posterior" would be the a distribution of lambda (given one or more observations y), not of the Poisson RV "y" as apparently used in the paper. (See for example https://stats.stackexchange.com/a/26225). | The term “posterior variance” was a misuse. Based on the comment **[2-3]**, “posterior variance” is now replaced with “an approximate variance for a log-transformed Poisson random deviate” |

| Comment from reviewer 3 | |
| --- | --- |
| Comment | Response |
| **[3-1]** The authors have carried out some commendable work on approximating the Poisson likelihood using a mode-matching log-Gaussian approach. However, the main motivation -- as mentioned in the abstract and introduction -- is on improved efficiency and accuracy for Bayesian Poisson regression, but Bayesian modelling does not appear anywhere in the paper. In all the Results section (including two simulations and the Japanese COVID-19 data analysis), the proposed approximation is only used for frequentist modelling via maximum likelihood and no Bayesian analysis is considered. Given that frequentist estimation and inference for Poisson regression with random effects/spatial effects is already very well developed via the MGCV and LME4 packages in R, the advancement using proposed approximation is minimised. | Thank you very much for the suggestive comment. We agree that our approximation is not a Bayesian approach. We also agree that frequentist estimation and inference for Poisson regression is already well developed.  Nevertheless, we believe that our proposed approximation is important because (i) it circumvents an identification problem in Poisson regression and (ii) it is much faster than the conventional Poisson regression.  Regarding (i) the identification problem, we added the following sentences (line 56-60):  The maximum likelihood estimates of the conventional Poisson regression are unidentifiable or identifiable only weakly for certain data configuration (Silva and Tenreyro, 2010; Correia et al., 2019). As we will illustrate later, this property considerably worsens the accuracy of Poisson regression estimates especially for small samples with many zeros. Their linear approximations are free from such difficulty and can be more stable.  After that, we showed in Figures 3-4, 6-7 that our approximation tends to have smaller RMSE and bias values for small samples (N=50) with many zeros (small β_0_).  Regarding (ii) the computational efficiency, we newly compared the computation time in the “Computation time comparison” section (line 269-279), The result confirms that our approximation is considerably faster than the conventional Poisson regression. |
| **[3-2]** Page 2 line 33: "corrected" = "collected" | “corrected” is replaced with “collected” |
| **[3-3]** Page 2 line 45: "contiguity" should be "conjugacy"? | Yes. “contiguity” is replaced with “conjugacy”. |
| **[3-4]** Page 3 Equation (2): the observation y appears on both sides of expression (2). It is mentioned in the following paragraph that "1/(y+c) approximates the posterior variance of the Poisson distribution" but what is this the posterior variance of? Is it the variance of the posterior distribution of P(lambda\|y)? | The term “posterior variance” was a misuse. It is now replaced with “an approximate variance for a log-transformed Poisson random deviate” based on a comment from another reviewer. |
| **[3-5]** Page 4 Equation (8): the expression here is not limited to over-dispersion. Some discussion (or simulations) based on under-dispersed counts would be interesting in this paper. | We agree with this comment.　We newly add the following sentence in line 369-371:  Our approach models under-dispersed count data if $\sigma^{2}<1$ whereas conventional Poisson models cannot consider under-dispersion. Application for under-dispersed data is another interesting topic. |
| **[3-6]** Page 6 line 135: how is the over-dispersed Poisson simulated in your paper? Did you use the Neg-Bin, or Poisson-Gamma, or some other simulation mechanism? | The following sentence is added to explain how to simulate the data (line 185-188):  Following Gsteiger et al. (2013), $y_{i}$ is sampled from a negative binomial distribution such that the assumption of the over-dispersed Poisson distribution (mean: $\lambda_{i}$, variance: $\lambda_{i}\sigma^{2}$) is fulfilled. |
| **[3-7]** Page 6 Equations (10): Would it be better to replace the RMSE by a relative RMSE, by dividing the squared difference by beta_k? | Because the coefficient estimation accuracy does not proportion to the scale of beta, we believe RMSE is more suitable in our case than relative RMSE. We decided to use (non-relative) RMSE as before. |
| **[3-8]** All the figures are very low resolution: I would recommend using a different image format for your figures. | We agree. We now replaced our image format to pdf and improved the resolution. |
