# Supplementary material for: Improved log-Gaussian approximation for over-dispersed Poisson regression: Application to spatial analysis of COVID-19
Source: PLoS One. 2022 Jan 7;17(1):e0260836. doi: 10.1371/journal.pone.0260836 (PMC8741021; doi:10.1371/journal.pone.0260836)
Supplement: Attachment — Submitted filename: draft_MM_v5_response.docx [file pone.0260836.s003.docx]

| **Comments from reviewer 2** | |
| --- | --- |
| Comment | Response |
| **[1]** Thank you for the major revision to lines 74 – 139. That makes it much easier to understand your derivation. Unfortunately, it also makes it possible to identify places where the derivation is not done carefully enough. Further comments on this are in the details. | Thank you very much for your suggestive comments below. We have revised our draft following the comments. |
| **[2]** Thank you also for including computing time information. That is helpful. | Thank you very much! |
| **[3]** I remain very concerned about the mismatch between the distributions assumed in the analysis and those used to simulate data. You cite Gsteiger et al to support using a negative binomial distribution to simulate data from the quasi-poisson distribution (i.e. where variance = lambda_i sigma^2). Where does Gsteiger say this? The text below equation (10) in section 2.2 very clearly says variance = mu + k mu^2, the usual expression for negative binomial variance. As I said in the first review, these are two very different variance patterns. | We agree the comment that this part is unclear. We newly added the following sentence to explain how to sample an over-dispersed Poisson variable using a negative binomial distribution (line 224-233 highlighted by yellow):  The simulation data is sampled to satisfy $E\left[ y_{i} \right]=\lambda_{i}$ and $Var\left[ y_{i} \right]=\sigma^{2}\lambda_{i}$, which $y_{i}\sim odPoisson\left( \lambda_{i},\sigma^{2} \right)$ assumes, as follows:   (a) Calculate $\lambda_{i}=\exp\left( \beta_{0}+x_{i,1}\beta_{1}+x_{i,2}\beta_{2} \right)$ (b) Calculate $v_{i}=\left( \sigma^{2}-1 \right)/\lambda_{i}$ (c) Sample $y_{i}\sim NB\left( \lambda_{i},v_{i} \right)$ where $NB\left( \lambda_{i},v_{i} \right)$ is a negative binomial distribution with expectation $\lambda_{i}$ and variance $Var\left[ y_{i} \right]=\lambda_{i}+v_{i}\lambda_{i}^{2}$   The sampled $y_{i}$ has the expectation $E\left[ y_{i} \right]=\lambda_{i}$ and variance $Var\left[ y_{i} \right]=\lambda_{i}+v_{i}\lambda_{i}^{2}=\lambda_{i}+\left( \sigma^{2}-1 \right)\lambda_{i}=\sigma^{2}\lambda_{i}$ that $odPoisson\left( \lambda_{i},\sigma^{2} \right)$ assumes. Thus, the sampled $y_{i}$ fulfills the assumption of the over-dispersed Poisson distribution.  Where does Gsteiger say this?  Gsteiger is not cited in the revised draft. |
| **[4]** I repeat my previous concern about “too small” SEs. A small SE is a good only when that se is correctly estimated. You claim you can’t evaluate the performance of the SE estimator. I don’t understand why not. If it is numerical issues, doesn’t this raise concerns about the practical use of your approximation. | Thank you for the suggestive comment. We consider that the too small SEs obtained from our method, is problematic. So, our approximation is modified partly to reduce the bias (see line 158-180; highlighted by light blue).  As newly demonstrated in Figures 5, SEs estimated from the modified method is close to the SEs obtained from over-dispersed Poisson regression.  Following a reviewer comment we got in the previous round, we also compare the SEs with the standard deviations of the estimated regression coefficients. Although the SEs still tend to be smaller than the standard deviation, the bias is smaller than the conventional Poisson regression and other Gaussian approximations (see Figures 6 and 10). |
| Specific details | |
| **[5]** Equation (2) doesn’t make probabilistic sense because y_i occurs in the specification of the distribution. Technically, you are giving the distribution of y_i conditional on y_i, which is meaningless. Similar issues occur through (6). A derivation could be done more carefully by using E y in the variance expression then substituting y_i as a “plug-in” estimator of E y_i at the end. That will clarify what is being assumed by the proposed approximation. Many do not like plug-in estimators, so you are free to find another way to derive your results that avoids a distribution of Y conditional on Y. | Thank you very much for the suggestive comment. We agree. To clarify what is being assumed, $E[y_{i}]=\lambda_{i}$ is used in the variance expression then $y_{i}$ is substituted as a plug-in estimator (lines 114-155).  We would like to leave extension of our method to not to rely on plug-in estimator for future research. The following sentence is added to explain this point (lines 155-156; highlighted by grey marker):  This plug-in method ignores the uncertainty in the variance term. Consideration of the uncertainty will be an important future task. |
| **[6]** Please check the derivation of equation (7). You seem to be using Var y^* = exp(mu + v), not exp(mu+v/2). The issue of y_i occuring in the variance expression is even more important here. | *Note: I response to this comment assuming “Var y^* = exp(mu + v), not exp(mu+v/2)” is correctly “E y^* = exp(mu + v), not exp(mu+v/2)” because exp(mu+v/2) represents the expectation of y, which has a log-Gaussian distribution.*  We checked the derivation of equation (11) in line 138, which was equation (7) in the previous version and confirmed that our derivation is correct. Equation (11) is derived from equation 10:  $\log\left( y_{i}^{*} \right)\sim N\left( \mu_{i}, \frac{1}{y_{i}+0.5} \right)$ (equation 10).  The expectation of $y_{i}^{*}$ equals  $E\left[ y_{i}^{*} \right]=\exp\left( \mu_{i}+\frac{v}{2} \right)$  which is suggested by the reviewer, where $v=\frac{1}{y_{i}+0.5}$. By multiplying $\exp\left( -\frac{v}{2} \right)$ on both side of the equation above, we have equation (11). |
| **[7]** L 125. Don’t you apply (6) with probability 1-r and (8) with probability r, where r is the proportion of 0 counts? | You are right. The corresponding part, which was wrong, is fixed as follows (line 148-149; highlighted by green marker):  Before: Eqs. (6) and (8) are applied with probabilities $r$ and $1-r$  After: Eqs. (10) and (11) are applied with probabilities $1-r$ and $r$ |
| **[8]** L 135: what happened to r in the definition of the response variable? This is probably a typo because the analogous expression in table 1 includes r. | Yes, it is a typo. $\log\left( y^{*} \right)=\log\left( \frac{y+0.5}{z} \right)-\frac{1.5}{y+0.5}$, which was in line 135, is now replaced with the following equation, which is consistent with table 1 (line 177; highlighted by pink marker):  $\log\left( y^{*} \right)=\log\left( \frac{y+0.5}{z} \right)-\frac{1+0.5r}{y+0.5}$ |
| **[9]** L 157: Yes, Laplace is often good but it doesn’t always work. There is a substantial literature on better approximations, e.g. numerical quadrature with more quadrature points. | We agree with this comment. The following sentence is added based on the comment (line 206-209; red text):  The Laplace approximation can have poor accuracy if the target distribution is far from Gaussian distribution. Extension based on other approximation methods such as numerical quadrature is an important remining task. |
| **[10]** L 203: Please be careful not to overstate your results. Estimates may be close on average, but that does imply they are accurate. I am especially concerned about B0, which seems (Figure 2) just as biased for N=200 as it is for N=50. If you consider an even simpler situation with a constant mean, inspection of equation (6) shows that estimates of B0 are not unbiased and not consistent, because of the approximation. | Thank you for the comment. We agree with the comment that the sentence “It is suggested that the proposed method estimates regression coefficients accurately”, which was in this part, is overstated. This sentence is removed. Besides, similar sentences, which were in the same section or later sections are removed or revised to not to make overstatement.  We also agree with the comment that the estimated B0 is not unbiased and not consistent. To overcome the problem, we modified our approximation (line 158-180; highlighted by light blue). The updated simulation results (e.g., Figures 1-4) demonstrate that the B0s estimated from our modified approximation has as small bias as the conventional Poisson regression. Besides, in case of over-dispersed Poisson regression, the bias of our method tends to be smaller than the conventional approach. |
| **[11]** L 210: What is sigma^2 here? I.e., what distribution is that a parameter of? If a negative binomial distribution is used to simulate data, I strongly suspect that data simulated with sigma^2 = 1 are still overdispersed, based on second major comment about variance models. | Please check the response to the comment **[3]**. I believe that the meaning of sigma^2 is now clear (sigma^2 is the dispersion parameter in the over-dispersed Poisson regression).  The following sentence is added to explain the meaning of sigma^2 here (line 279-280; green text):  $\sigma^{2}=5$, which means an overdispersion with variance $Var\left[ y_{i} \right]=5\lambda_{i}$ |
| L 315: overall, age-group is much clearer than generation. Thank for changing that wording. Here is one lingering use of generation. | Thank you very much! |
